# Tuberculosis of Man and Cattle

**Published:** 1901-08

**Authors:** 


### TUBERCULOSIS OF MAN AND CATTLE.

Professor Koch, the great German bacteriologist and the discoverer of the tubercle bacillus, has recently read a paper at the Tuberculosis Congress in London in which he has announced the opinion that there is little danger of the transmission of tuberculosis to man by the food products of tubercular cattle. Strange to say, this opinion is not based on new or original observations, but is drawn from facts that have been the common property of the scientific world for years, although they have not heretofore been interpreted by any bacteriologist or pathologist as Koch has interpreted them.

Everyone must wish that Koch is correct, and if he is the problem of suppressing tuberculosis in herds will be vastly simplified. The problem will still be serious, but it will be important from but one point of view—*i. e.*, from that of the owner of cattle. It will then be on the basis of contagious pleuropneumonia, cattle plague, Texas fever, hog-cholera, etc.—diseases that are confined to one or more species of farm animals and are not transmissible to man.

To check the spread of tuberculosis in a herd it is only necessary to separate the non-tubercular from the tubercular cattle, keep them in uncontaminated quarters, and give their food free from tubercle bacilli.

By the use of the tuberculin-test it is now comparatively easy to make this separation. At present it is not deemed safe to sell for human consumption the uncooked milk of tubercular cattle. If Koch's view shall prevail such milk may be sold freely and without restriction. In this event the owner of a tubercular herd will suffer little loss through the application to his herd of measures to repress tuberculosis. The market for his dairy products will not be interfered with.

But veterinarians must not lose sight of the fact that tuberculosis has been known in hundreds, yes, thousands, of cases to have been transmitted in the milk of tubercular cows to calves and swine. This danger is so evident that in some countries it is required by law that the skim milk returned to farms from creameries shall be pasteurized, and where this has been done there is a diminution in the prevalence of tuberculosis among calves and swine. So, even if Koch is right, we must be careful not to allow the unheated milk from tubercular cows to be fed to calves or swine, even though it may be fed freely to invalids and babies.

Another possible effect of Koch's dictum, admitting for the moment that he is right, will be the removal of veterinary sanitary work from boards of health, where it is so vested, to veterinary boards, live-stock sanitary boards, cattle commissions, etc.

But let us examine the evidence upon which Koch's opinion is based. No opinion upon a scientific subject is any better than the facts upon which it is grounded. Science is not controlled by opinions, but by facts. Koch's facts are two: 1. He has observed that human tuberculosis is not readily inoculable upon cattle. From this he concludes that cattle tuberculosis is not readily inoculable upon man. 2. He has noted the comparative rarity of what he terms primary intestinal tuberculosis, and from this concludes that infection by way of the digestive tract is so exceptional as to be of little importance.

The first question is, Is the bovine tubercle bacillus virulent for man? This question cannot be determined by deliberate inoculation of a human being, but enough inoculations have taken place accidentally to show that the bovine tubercle bacillus finds in the human tissues the conditions it requires for its growth, and that it is capable of causing the death of persons so inoculated.

Moreover, comparative tests of the virulence of human and bovine tubercle bacilli have been carried out on a large scale, and these, as made by Smith, Dinwiddie, Frothingham, and by Pearson and Ravenel, show that bovine tubercle bacilli are uniformly more virulent for all species of animals upon which they have been tested than are the human tubercle bacilli.

These investigations furnish reason to believe that the bovine bacillus is a more virulent germ throughout, and it is quite within the range of possibility that it is also more virulent for man than the bacillus that has been grown for some time in human tissues.

As to the second point: the rarity of primary intestinal tuberculosis. It is erroneous to assume that the solution of this question can be found in the distribution of the lesions of tuberculosis in the body. It has been shown in the most unmistakable way by many feeding experiments conducted under the auspices of the Pennsylvania State Live-stock Sanitary Board that, contrary to the early belief, animals fed tubercular material may develop primary pulmonary tuberculosis, and, in some instances, fail to show lesions in any other organs. It is strange that Prof. Koch failed to observe the importance of this point, for in his address he says: "Among the animals [swine] that had been fed with sputum no trace of tuberculosis was found, except here and there little nodules in the lymphatic glands of the neck, and, in one case, a few gray nodules in the lungs." In these cases the animals were infected by feeding, but did not develop tuberculosis in the intestines first or at all. A little later he says: "The animals that had eaten bacilli of bovine tuberculosis have without exception (just as in the cattle experiment) severe tuberculous diseases, especially of the lymphatic glands of the neck and of the mesenteric glands, and also *extensive tuberculosis of the lungs* and spleen." Here, again, is pulmonary tuberculosis from feeding complicated, it is true, by disease in other organs; but who can say, without knowledge of the history, by what channel the bacilli entered?

Moreover, it is shown by the statistics of the General Register Office of England, as quoted by the Royal Commission on Tuberculosis, which reported to Parliament in 1898, that "for the age-period 0 to 5 years there has also been a reduction in death from tubercular disease in all forms from 5764 per million living at that age in 1851 to 1860 to 4155 in 1891 to 1895, a diminution of 27.9 per cent. This reduction is, however, but little more than half that which has taken place in the age-periods fifteen to twenty years and twenty to twenty-five years; and when we come to examine that form of tubercular disease, namely, tabes mesenterica, which is believed to be mainly due to infection received by the digestive tract, we find that while at the age-period of 0 to 5 years, when milk forms so important an article of diet, the rate of death per million living has shown first increase, then decrease, yet the rate which stood at 1625 during 1851 to 1860 had only fallen to 1577 for the period of 1891 to 1895—a diminution of but 3 per cent. In this connection we feel it necessary to recall the fact that the term tabes mesenterica as used in our death records is very indefinite, and that it has comprised, and doubtless still comprises, a considerable amount of death from certain diarrhœal affections which are not really tubercular. But while this is so, it must also be remembered that the rate of mortality from tabes mesenterica which, more than any other, represents tuberculosis in infancy, has signally failed to undergo any noteworthy diminution during the very period of sanitary progress which has been associated with such substantial diminution of death from tubercular affections at all ages in England and Wales, and that this result has coincided in point of time with a large increase in the consumption of milk."

Moreover, at one period of life, before the end of the first year, when the child is on an almost exclusive milk diet, there has been an actual increase of tabes mesenterica of not less than 27.7 per cent. (Sir Richard Thorne.)

If children contract tuberculosis solely, or even chiefly, from adults, one would expect the prevalence of tuberculosis among them to fall in a ratio proportional to the ratio of diminution of tuberculosis among their elders. Since this is not the case, and since houses are much improved as to sanitary conditions, it is strongly indicated that children are infected with tuberculosis from some source outside of their families and homes.

With such facts before it the British Congress on Tuberculosis has done well not to accept Koch's view. It has recommended the continuance of all public and private measures for the repression of tuberculosis of cattle, and advised a thorough scientific review of the whole subject of the relation between human and bovine tuberculosis.

